# Monitoring endocrine-disrupting chemicals and microbial diversity of wastewater treatment maturation ponds

**DOI:** 10.1128/aem.01342-25

**Published:** 2026-04-29

**Authors:** L. B. Qhanya, A. O. Ojo, G. Kemp, E. D. Cason, O. de Smidt

**Affiliations:** 1 Centre for Applied Food Sustainability and Biotechnology (CAFSaB), Central University of Technology 71795, Bloemfontein, Free State, South Africa; 2 Department of Microbiology and Biochemistry, University of the Free State 37702, Bloemfontein, Free State, South Africa; 3 Department of Animal Science, University of the Free State 37702, Bloemfontein, Free State, South Africa; Washington University in St Louis, St. Louis, Missouri, USA

**Keywords:** wastewater stabilization ponds, wastewater effluent, maturation ponds, endocrine-disrupting chemicals, emerging pollutants

## Abstract

Endocrine-disrupting chemicals (EDCs), including synthetic hormones, pesticides, and personal care products (PPCPs), are frequently detected in wastewater treatment plant (WWTP) effluents. EDCs’ persistence in aquatic environments poses risks to human and ecological health, given that EDCs are not firmly regulated under South African legislation. The chemical and microbial compositions of a WWTP utilizing maturation ponds in Bloemfontein, South Africa, focusing on selected EDCs (atrazine [ATZ], 17α-ethinylestradiol [EE2], triclosan [TCS], and bisphenol A [BPA]) were investigated. The physicochemical parameters of wastewater samples collected over 6 months were measured onsite. LC-MS/MS analysis and bacterial 16S rRNA and eukaryotic sequencing were performed to determine the EDC concentrations and microbial compositions. Wastewater samples exhibited alkaline, oxic, mesophilic, and oxidative conditions. The EDC concentrations showed variability, with 17α-ethinylestradiol reaching 125 µg/L. Bisphenol A concentration ranged between 0.003 and 0.587 µg/L, that of atrazine from 0.009 to 0.134 µg/L, and that of triclosan from 0.069 to 0.316 µg/L, which were lower than those reported in other South African studies. Microbial community structures revealed Proteobacteria (35.12%) and Bacteroidetes (18.50%) as the dominant bacterial phyla. Among eukaryotes, Ascomycota and unclassified taxa accounted for 13.14% and 68.85%, respectively. *Flavobacterium* and *Microcystis* were the most abundant bacterial genera (9.50% and 6.22%), while unidentified taxa represented 85.88% of eukaryotic genera. Functional analysis revealed potential xenobiotic degradation pathways associated with bisphenol A and atrazine, indicating microbial potential for EDC degradation. These findings established the presence of EDCs and microbial compositions of maturation ponds with an estimated hydraulic retention time of ~9 days, emphasizing the need for improved wastewater treatment strategies and bioremediation approaches.

**IMPORTANCE:**

Endocrine-disrupting chemicals (EDCs) are increasingly found in treated wastewater, yet their removal is not guaranteed in conventional treatment systems. This study examined maturation ponds of South African wastewater treatment plant and provided new insights into their chemical pollutant retention and microbial dynamics. By tracking selected EDCs together with bacterial and eukaryotic communities, the study showed how environmental conditions and microbial populations interact within the ponds. The detection of microbes with potential to degrade pollutants highlighted a natural capacity that could be strengthened for bioremediation. South Africa relies heavily on treated wastewater as a supplementary water source, and understanding the presence of EDCs and the biological process within these ponds is essential. These findings contribute to improving wastewater treatment practices and developing sustainable, microbially driven solutions for safer water management.

## INTRODUCTION

In recent years, rapid industrialization and urbanization have released numerous unregulated chemical and biological substances into the environment, commonly known as emerging pollutants (EPs) (1). These pollutants come from various sources, including industrial processes, agriculture, pharmaceuticals, and urban activities. EPs encompass over 20 classes of compounds, including persistent organic pollutants (POPs), pharmaceuticals and personal care products (PPCPs), veterinary drugs such as antibiotics, endocrine-disrupting chemicals (EDCs), and nanomaterials such as micro- and nanoplastics. Despite their growing presence, much remains unknown about their environmental behavior and potential impacts (2, 3). Endocrine-disrupting chemicals (EDCs) are frequently detected in water bodies, including effluent, influent, and sludge of wastewater treatment plants (WWTPs).

The sources of EDCs in the environment include municipal and household wastewater, building materials, agricultural runoff, mining, industrial emissions, and solid waste. These pollutants are often not effectively removed by wastewater treatment plants (WWTPs), resulting in their entry into water bodies, where they are taken up by various organisms (4) (5) (6). EDCs include synthetic hormones such as 17α-ethinylestradiol (EE2), triclosan (TCS), atrazine (ATZ), and bisphenol A (BPA); these compounds enter the environment through wastewater (7). EE2 is found in birth control pills and is the most widely used contraceptive (8), while triclosan, a broad-spectrum antimicrobial, is a key component in many pharmaceuticals, personal care products, and household items such as bedding, dishwashing products, and sporting goods (7). Atrazine, once the most widely used herbicide against broadleaf weeds, was banned in the USA and Europe in 2003 due to its presence in drinking water (9). Bisphenol A is another common EDC found in a wide range of plastic consumer products, including the linings of tin cans, food and water containers, medical devices, and toys (10). These compounds persist through conventional treatment processes, posing a significant challenge to environmental and public health due to their potential to interfere with hormonal systems even at low concentrations (11–13). Unlike traditional pollutants, EDCs tend to be persistent and stable in the environment, such as water bodies, at low concentrations (14). For instance, EDCs were detected in four rivers in the Eastern Cape, South Africa, with average concentrations of 0.1471 ug/L (bisphenol A), 0.7334 ug/L (triclosan), 0.0285 ug/L (atrazine) (15), and 0.004 ug/L (EE2) (16).

The emerging pollutants, including the EDCs, in surface water are a global concern due to their potential adverse effects on human health and ecosystems (17, 18). The increase in endocrine-related diseases in female and male reproductive system disorders and cancers, particularly breast, endometrial, ovarian, cervical, prostate, and testicular cancers, has heightened public concern about the EDCs’ roles (19–21). For instance, a widespread EDC, such as BPA, found in many consumer products, has been linked to an increased risk of certain cancers (22) and shown to negatively affect female reproductive health, including fertility (23). EE2 has been linked to endocrine disruption in aquatic species, causing feminization and reproductive issues, with some wastewater effluent concentrations exceeding safe levels for aquatic organisms (16). The detection of these pollutants in surface waters underscores the need for ongoing monitoring, comprehensive risk assessments, and effective mitigation strategies to protect human and environmental health (24–27). These contaminants often co-exist at polluted sites (28–31), and developing methodologies for their simultaneous and efficient degradation would be valuable. While chemical and physical remediation techniques can offer rapid results, microbial degradation is eco-friendly and cost-effective for removing EDCs from contaminated environments under anaerobic or aerobic conditions (32).

Most previous biodegradation studies have primarily focused on isolated pure cultures (33–35). Aerobic bacteria belonging to Proteobacteria, Actinobacteria, and Firmicutes phyla were identified as efficient EDC degraders (36). Among eukaryotes, EDC-degrading agents were found within genera such as *Phanerochaete chrysosporium*, *Trametes versicolor*, *Bjerkandera adjusta,* and *Pleurotus* sp (37, 38). Presently, more than 80 bacterial genera are classified as EDC-degraders; these include species of *Bacillus*, *Virgibacillus*, *Novosphingobium*, *Rhodococcus*, *Acinetobacter*, *Pseudomonas*, *Sphingomonas*, *Enterobacter*, *Klebsiella*, *Aeromonas*, *Comamonas*, *Thauera*, *Deinococcus*, *Stenotrophomonas,* and *Mycobacterium* (39, 40). However, these isolates may not be the dominant degraders within contaminated environments (41–43). Microbial consortia, with their broad enzymatic capabilities, offer an advantage over individual microorganisms due to synergistic interactions within microbial communities, leading to more effective and robust degradation of contaminants (44). Recent advancements in high-throughput sequencing technologies have greatly enhanced the ability to investigate microbial ecology in environments contaminated with EDCs and their metabolic potential in degrading these contaminants.

Recently, attention has grown around characterizing enriched consortia capable of degrading various contaminants (45, 46). Understanding the ecology and phylogeny of indigenous microbial consortia from diverse contaminated sites is essential for devising effective clean-up strategies. However, the use of enriched consortia for remediating mixed EDCs remains in its early stages. Questions remain about how major degraders vary when growing on different contaminants and how degradation efficiency shifts with various initial microbial sources. The present study examined the microbial community structure and metabolic potential associated with EDC degradation in wastewater collected from maturation ponds. The objectives were to (i) gain an overview of the pond systems’ physicochemical characteristics, (ii) quantify the concentrations of selected EDCs in the maturation ponds, (iii) identify the microbial composition within the pond system, and (iv) predict the functional potential of indigenous microorganisms that could be responsible for EDC removal.

## MATERIALS AND METHODS

High purity (>98%) chemical standards for bisphenol A (BPA), atrazine (ATZ), 17α-ethinylestradiol (EE2), and triclosan (TCS) were purchased from Sigma Aldrich (St. Louis, MO, USA), while internal standards (positive mode: atrazine-d5 and negative mode: bisphenol A-d16) were provided by Facility for Genomics and Proteomics, Microbial, Biochemical and Food, University of the Free State. The stock solution for each standard was prepared in methanol at a concentration of 1 μg/L. High-performance liquid chromatography (HPLC)-graded methanol (MeOH), formic acid, and ammonium hydroxide were purchased from Sigma-Aldrich (St. Louis, MO). Ultra-pure water (18 Ω) prepared using a Milli-Q purification device (Millipore, Billerica, MA, USA) was used in all experiments.

### Sampling sites and sample collection

**Fig 1:**
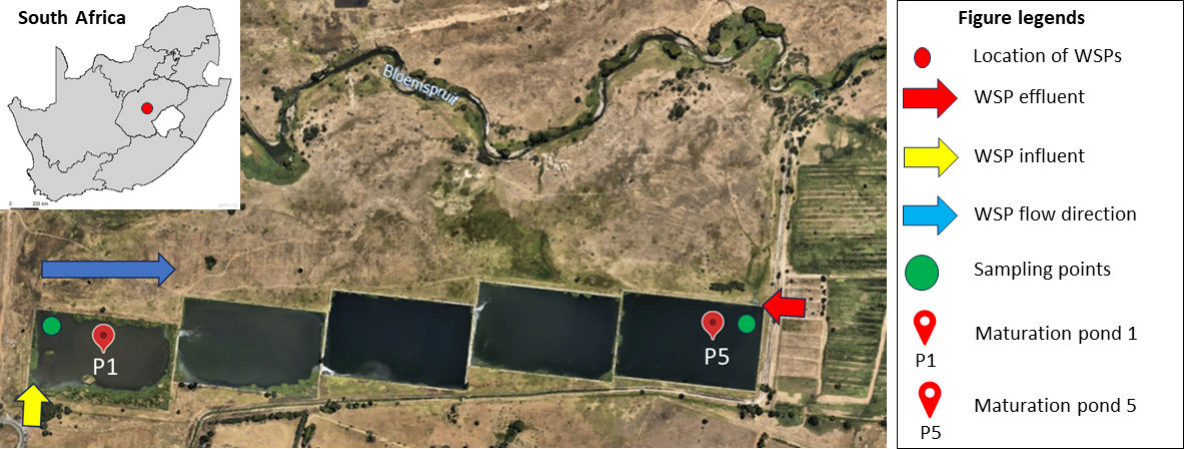
Sampling sites and the configuration of the maturation pond system. P1, inflow; P5, outflow. Imagery©2026 Google, Imagery©2026 Airbus, CNES/Airbus, Maxar Technologies, Map data©2026 AfriGIS (Pty) Ltd.

Six samples were collected from each of P1 and P5 of a wastewater stabilization pond (WSP) system comprising five maturation ponds over 6 months during spring and summer (Fig. 1). This WSP is the only facility in Bloemfontein, South Africa, that still operates a series of wastewater stabilization ponds as part of its treatment system. The maturation pond system operates sequentially, receiving treated wastewater from the Bloemspruit WWTP into Pond 1 (P1). From there, the water flows by gravity through the series of ponds, undergoing further natural treatment until it reaches Pond 5 (P5), where the final effluent is discharged into the Bloemspruit stream. The system is situated a few kilometers (~1.7 km) from the Bloemspruit wastewater treatment plant (WWTP), Bloemfontein, South Africa. The ponds serve a population of approximately 2,834,714 people and receive 57,000 m³/d of wastewater per day (47). Samples from Ponds 1 (inflow [influent]: 29°07’32’’S; 26°16’32’’E) and 5 (outflow [effluent]: 29°07’32’’S; 26°16’06’’E) were collected into 1 L pre-washed and sterilized amber (Simax) glass bottles and secured with screw caps. Bottles were rinsed twice with the respective pond samples before filling, and all the samples were collected 1 meter from the surface using a grab sampling stick. Samples were stored in ice, transported to the laboratory for further analysis, and analyzed within 12 h after sampling.

### Chemical analysis

#### Physicochemical parameter analysis

To determine the overall quality of the maturation ponds and assess the effectiveness of wastewater treatment, parameters such as temperature, pH, electrical conductivity (EC), and total dissolved solids (TDS) were measured with a portable multiparameter field meter (HACH SENSION + MM150, USA), and dissolved oxygen (DO) and oxidative reduction potential (ORP) were measured on-site with a portable multiparameter meter HACH HQ40D.

#### Liquid chromatography-tandem mass spectrometry (LC-MS/MS) analysis

Samples were analyzed using a Sciex 4000 QTRAP hybrid triple quadrupole ion trap mass spectrometer with a Shimadzu HPLC stack as a front end. All data acquisition and processing were performed using Analyst 1.5 software. Twenty microliters (20 µL) of each extracted sample, standard, blank, and control were analyzed twice: once in the positive ionization mode for ATZ and once in the negative ionization mode for TCS, BPA, and EE2. The targeted analyses were performed using two MRM (multiple reaction monitoring) transitions. The peak area on the chromatogram generated from the first and most sensitive transition was used as the quantifier, while that from the second transition was used as a qualifier. The qualifiers serve as an additional level of confirmation for the presence of the analyte, and the retention time for these two transitions needs to be the same. Table S1 shows data for relevant HPLC and mass transitions for each analyte. In the positive mode, the sample was separated on a C18 column at a flow rate of 300 uL/min using a 4 min gradient from 5% solvent A to 95% solvent B. The total run time was 12 min to allow for column re-equilibration after the gradient. Eluting analytes were analyzed in the positive ionization mode in the TurboV ion source with 500°C heater temperature to evaporate excess solvent, 35 psi nebulizer gas, 50 psi heater gas, and 50 psi curtain gas. The ion spray voltage was set at 5,500 V. The samples with analytes favoring negative ionization were separated on C18 columns at a flow rate of 300 uL/min using a 4 min gradient from 5% solvent A to 95% solvent B. The total run time was 12 min to allow for column re-equilibration after the gradient. Eluting analytes were analyzed in the negative mode and ionized by electrospray in the TurboV ion source with 500°C heater temperature to evaporate excess solvent, 35 psi nebulizer gas, 50 psi heater gas, and 50 psi curtain gas. The ion spray voltage was set at 4,500 V for the negative mode and 5,500 V for the positive mode. A six-level calibration curve was generated by spiking 500 mL of purified lab water with each analyte, ranging in concentration from 10 ppm (part per million; mg/L) to 0.0001 ppm, after SPE extraction with a linear fit through the origin, producing a correlation coefficient (r-value) in excess of 0.98. Blank samples were included before analysis of the extracted samples to ensure no background levels of the analytes.

### Microbial characterization

#### Genomic DNA extraction, PCR amplification, and sequencing

Prior to the DNA extraction, 1 L of each sample was centrifuged in a centrifuge (Neofuge 15R) at 5,000 × *g* for 5 min, and the supernatant was discarded. The obtained pellets were transferred to a 2 mL microcentrifuge tube, and genomic DNA extraction was performed using the procedure described in the NucleoSpin DNA Stool Kit (Macherey-Nagel GmbH & Co. KG, Düren, Germany). DNA concentration and quality were assessed using a NanoDrop 2000 spectrophotometer (Thermo Scientific, NanoDrop products, Wilmington, DE, United States) and 0.8% agarose gel electrophoresis. Each DNA extract was standardized to 100 ng/L in nuclease-free water (Whitehead Scientific, Cape Town, South Africa). Genomic library preparation and sequencing were performed at the Agricultural Research Council (ARC)–Onderstepoort Veterinary Institute in Pretoria, South Africa. A directed approach was used to obtain the bacterial 16S rRNA amplicon sequencing library by amplifying a ∼550 bp region located in the hypervariable V3 and V4 region of the 16S rRNA gene using region of interest-specific primers with overhang Illumina adapter overhang nucleotide sequences (16S F 5′–TCGTCGGCAGCGTCAGATGTGTATAAGAGACAGCCTACGGGNGGCWGCAG–3′ and 16S R 5′–GTCTCGTGGGCTCGGAGATGTGTATAAGAGACAGGACTACHVGGGTATCTAATCC–3′) (48). Eukaryotic community sequencing libraries were obtained by amplification of the internal transcribed spacer 1 (ITS1) region using ITS1 and ITS2 primers with Illumina adapter overhang nucleotide (ITS1 5′–TCGTCGGCAGCGTCAGATGTGTATAAGAGACAGTCCGTAGGTGAACCTGCGG–3′ and ITS2 5′–GTCTCGTGGGCTCGGAGATGTGTATAAGAGACAGGCTGCGTTCTTCATCGATGC–3′) (49). The amplicons were purified using the Agencourt AMPure XP bead clean-up kit (Beckman Coulter Genomics, Danvers, MA, United States), followed by a second amplification to attach dual indices and Illumina sequencing adapters using the Nextera XT Index kit (Illumina, San Diego, CA, United States). Final purification using the AMPure XP bead clean-up kit (Beckman Coulter Genomics) was performed, followed by library quantification, normalization, pooling, and denaturing before being subjected to 2 × 300-cycle sequencing on the Illumina MiSeq using the MiSeq v3 reagent kit (Illumina). Sequences obtained were deposited into the National Center for Biotechnology Information (NCBI) Sequence Read Archive (SRA) database. The accession number for all bacterial and eukaryotic sequences deposited into the NCBI-SRA database is PRJNA1234587.

#### Data processing and statistical analysis

The raw sequences obtained were analyzed using Quantitative Insights into Microbial Ecology v.2, QIIME2 (https://qiime2.org) (accessed 12 August 2023) (50). The paired-end reads imported into QIIME2 were quality-controlled and combined using DADA2. This resulted in amplicon sequence variants (ASVs) with 99% similarity. The ASV table was built, and chimeras were removed. The taxonomy for the bacterial 16S rRNA and eukaryotic sequences was assigned using the SILVA (v132) (51) and UNITE (v8.20) (52) databases. The alpha rarefaction curve was constructed with 1,000 iterations, which showed that the diversity present in all samples was adequately captured. To account for differences in sequencing depth among samples, all data sets were rarefied to the minimum sequencing depth across the data set (51,682 reads per sample) prior to diversity analyses.

The R-Studio (53), an Integrated Development Environment (IDE) for programming the R language (54), was used to analyze the processed gene data further (55). Homogeneity of variances for physicochemical parameters among seasons was assessed using Bartlett’s test (*P* > 0.05), confirming that the assumption of equal variances was met. Preliminary analyses indicated no significant differences in physicochemical parameters across seasons (ANOVA, *P* > 0.05). Therefore, seasonal samples were pooled and treated as replicates in subsequent analyses to increase statistical power. Paired *t*-tests were used to determine the significant differences in the physicochemical parameters between the inflow and outflow of the maturation pond system. Alpha diversity indices (Shannon, Observed, and Chao1 test) were calculated using the Vegan R package (56), and Bray–Curtis distance was used to generate a dissimilarity matrix. The microbial community structure was visualized using principal coordinate analysis (PCoA). The *Adonis* function in Vegan for the R package was used to perform a permutational analysis of variance (PERMANOVA), which was used to test the relationship between community composition and continuous environmental variables (pH, EC, and TDS) based on Bray–Curtis dissimilarities between the ponds. Preliminary analyses indicated no significant differences in microbial diversity across seasons (PERMANOVA, *P* > 0.05). Therefore, seasonal samples were pooled and treated as replicates in subsequent analyses to increase statistical power. Differences within habitats/ponds were further tested using the permutational homogeneity test of multivariate dispersions (PERMDISP) with the *betadisper* function from the R package Vegan v2.4-1. *Capscale* function from the R package Vegan v2.4-1 was used to perform distance-based redundancy analysis (db-RDA) to summarize the variation in microbial community structure in response to physicochemical parameters and EDCs.

### Functional analysis

To obtain insights into the functional potential of the bacterial communities and identify putative metabolic pathways related to energy metabolism as well as the degradation and metabolism of xenobiotic compounds, specifically drug metabolism pathways, taxon-based metabolic profiling using the PICRUSt2 pipeline (57), as implemented in QIIME2, was performed with default settings. As a prerequisite for PICRUSt2 analysis, taxonomy classification was redone using reference sequences from the Greengenes 13_8 99% database (58). The functional metagenome prediction data were analyzed using the “categorise by function” command (level 3) in the Kyoto Encyclopedia of Genes and Genomes (KEGG) pathways. Only KEGG pathways in energy metabolism and the degradation and metabolism of xenobiotic compounds, specifically drug metabolism pathways, were considered in the data analysis. The ASVs with the highest gene count contribution values for these pathways were determined for WSP samples.

## RESULTS AND DISCUSSION

### Physicochemical parameters of WSP maturation pond (inflow and outflow ponds)

**Fig 2:**
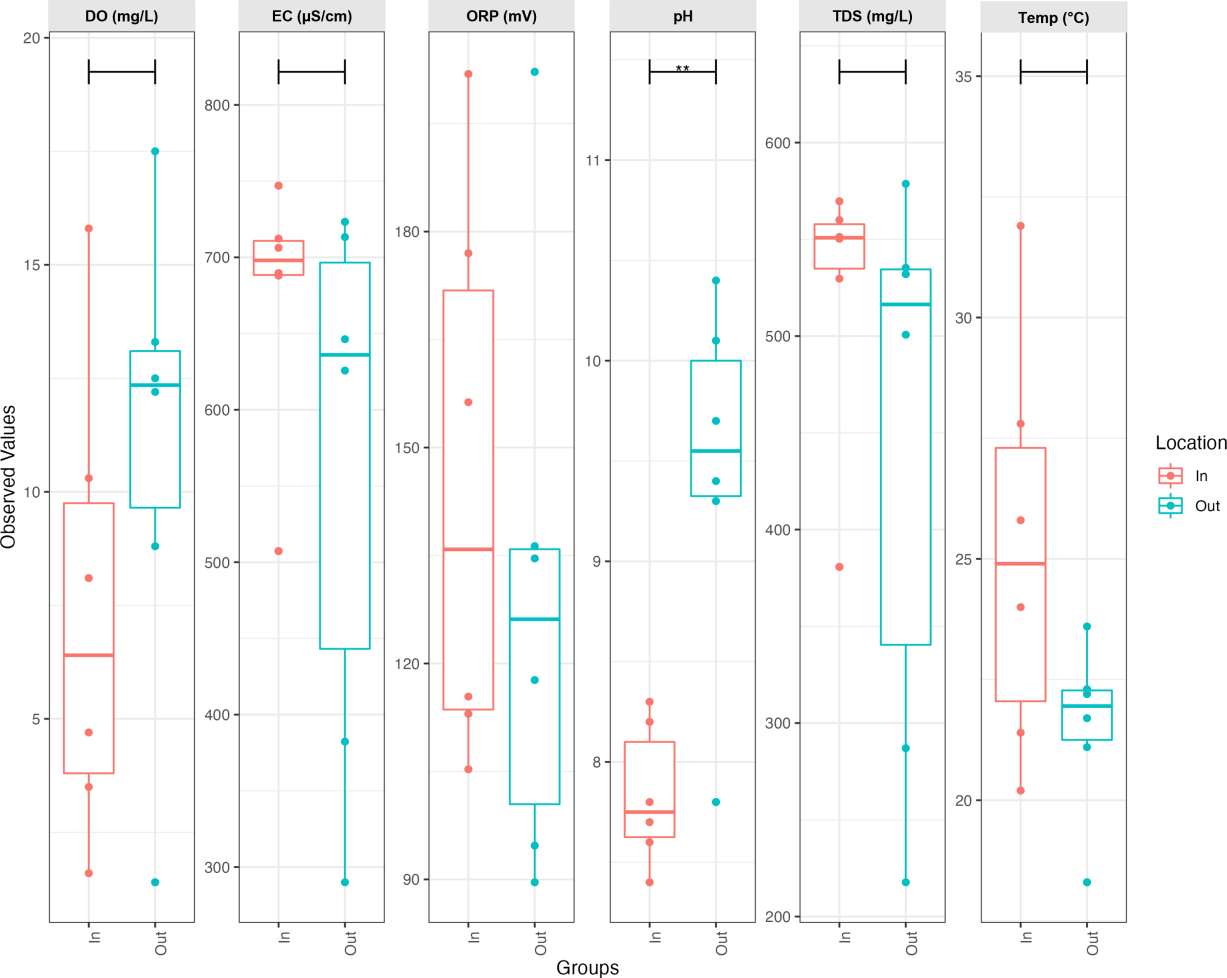
Boxplot of physicochemical parameters during the sampling period between the inflow (red boxplot In) and the outflow (blue boxplot Out) ponds of the maturation pond system (** *P* < 0.01) (*n =* 3).

Physicochemical analysis during the sampling period of 6 months indicated alkaline (inflow pH: 7.85 and outflow pH: 9.45), oxic (inflow DO: 7.18 mg/L and outflow: 10.81 mg/L), mesophilic (~25°C and ~21°C for inflow and outflow ponds), and oxidative (ORP of 144.80 mV [inflow] and 129.81 mV [outflow]) conditions (Fig. 2; Table S2). There was a significant difference in the physicochemical parameters of inflow and outflow ponds (*P* < 0.01). There was a notable increase in pH and DO concentrations from the inflow to the outflow, while the ORP concentration and temperature of the inflow were higher than those of the outflow. The pH values obtained in this study were higher than those of 6.10 to 7.03, as reported by Igbinosa et al. (59) during the assessment of treated wastewater effluents in a rural South African community. The allowable pH range for effluent discharge into the environment is 5.5 to 9.5 (60). As such, the mean pH values of the maturation ponds are within the stipulated allowable level.

The mean temperatures of the inflow and outflow ponds were ~25.2°C and ~21.5°C, respectively (*P* < 0.05). This range of temperatures supports optimal microbial activity, including efficient decomposition or degradation of chemical compounds (61). Theoretically, a linear relationship exists between EC and TDS, where high EC values typically correspond to high TDS. However, factors such as temperature and the presence of certain dissolved solids can influence the TDS and EC ratio. As such, EC and TDS were measured independently. The electrical conductivity (EC) mean values were 675 µS/cm (inflow) and 563 µS/cm (outflow), indicating a low level of dissolved solids and ions. These EC values are below the stipulated maximum limit of 15,000 µS/cm (62) for effluents to be discharged into the water bodies. The mean total dissolved solids (TDS) of the inflow and outflow were ~524 mg/L and ~442 mg/L, respectively. This indicates that the effluent from the outflow pond is suitable for irrigation since TDS levels below 700 mg/L are considered safe for irrigation (63).

### LC-MS/MS analysis

**Fig 3:**
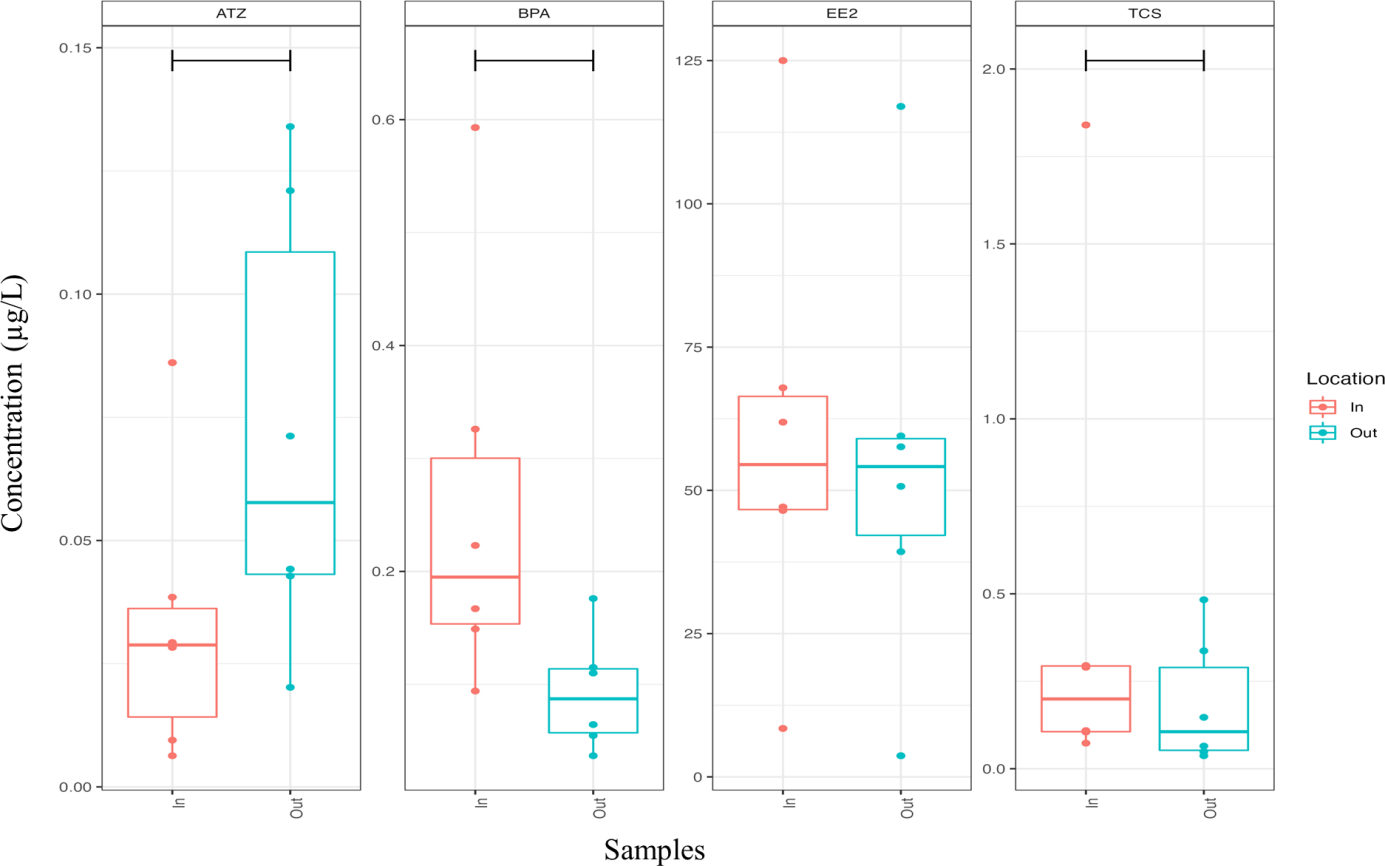
Box and whisker plots of EE2, TCS, BPA, and ATZ concentrations between the WSPs. Red boxplot In, inflow; blue boxplot Out, outflow ponds (*n =* 3).

During the sampling period, all four selected EDCs were detected regularly in both the inflow and outflow pond samples at varying concentrations (Fig. 3). The BPA concentration in the inflow pond varied from 0.089 to 0.587 µg/L, while a range of 0.003 to 0.145 µg/L was observed in the outflow pond. A study conducted in and around Cape Town, South Africa, reported BPA concentrations of 12.66 ± 0.81 µg/L in Bellville maturation pond effluents (64). This BPA concentration is higher than the study’s inflow and outflow BPA concentrations. This reported concentration is higher than those observed in both the inflow and outflow ponds of the present study. The maximum contaminant level of BPA is yet to be firmly established in South Africa, the European Union, or the United States.

ATZ concentration ranged from 0.009 µg/L to 0.134 µg/L between inflow and outflow ponds. A comparable concentration of 0.0095 µg/L of ATZ in Uitenhage-treated effluents in the Eastern Cape, South Africa, was reported (15). In 2016, the Environmental Protection Agency (EPA) conducted an atrazine ecological risk assessment, which determined that the scientifically derived concentration of atrazine, measured as a 60-day average, adversely impacts the aquatic environment at 3.4 µg/L. However, considering the Scientific Advisory Panel (SAP) recommendation, the EPA recalculated the level of concern for atrazine as 9.7 µg/L as a 60-day average (65).

Although the use of atrazine has been banned in Germany since 1991 (66), the EU has established Environmental Quality Standards (EQS) for atrazine, with an annual average of 0.6 µg/L and a maximum allowable level of 2 µg/L (67). Atrazine concentrations in the inflow and outflow ponds were below the stipulated EU and US standards. The concentration of EE2 in the inflow pond ranged from 0.0057 µg/L to 125.0 µg/L, and a range of 0.009 µg/L to 117.0 µg/L was detected in the outflow pond. In comparison, a maximum EE2 concentration of 0.008 µg/L in South African wastewater effluent, which is considerably lower than the values observed in the current study, was reported (68).

The mean concentrations of TCS detected in the inflow pond and the outflow pond were 0.316 μg/L and 0.069 μg/L, respectively. These observed concentrations were lower than the 0.09 ± 0.12 µg/L concentration in South African municipal wastewater reported by Kanama et al. (69). EE2 and TCS are listed on the EU watchlist, whereas the US and South Africa have not established standards for these compounds in water.

### Bacterial diversity and abundance

A total of 5,177 bacterial ASVs, ranging from 190 to 705 per sample, were identified with >99% sequence identity. Of the total number of ASVs, 2,771 (representing 8.24% of the total number of sequences) were unique to inflow samples, 2,454 (7.17%) were unique to outflow samples, and 2,455 (84.58%) were shared between all the locations.

**Fig 4:**
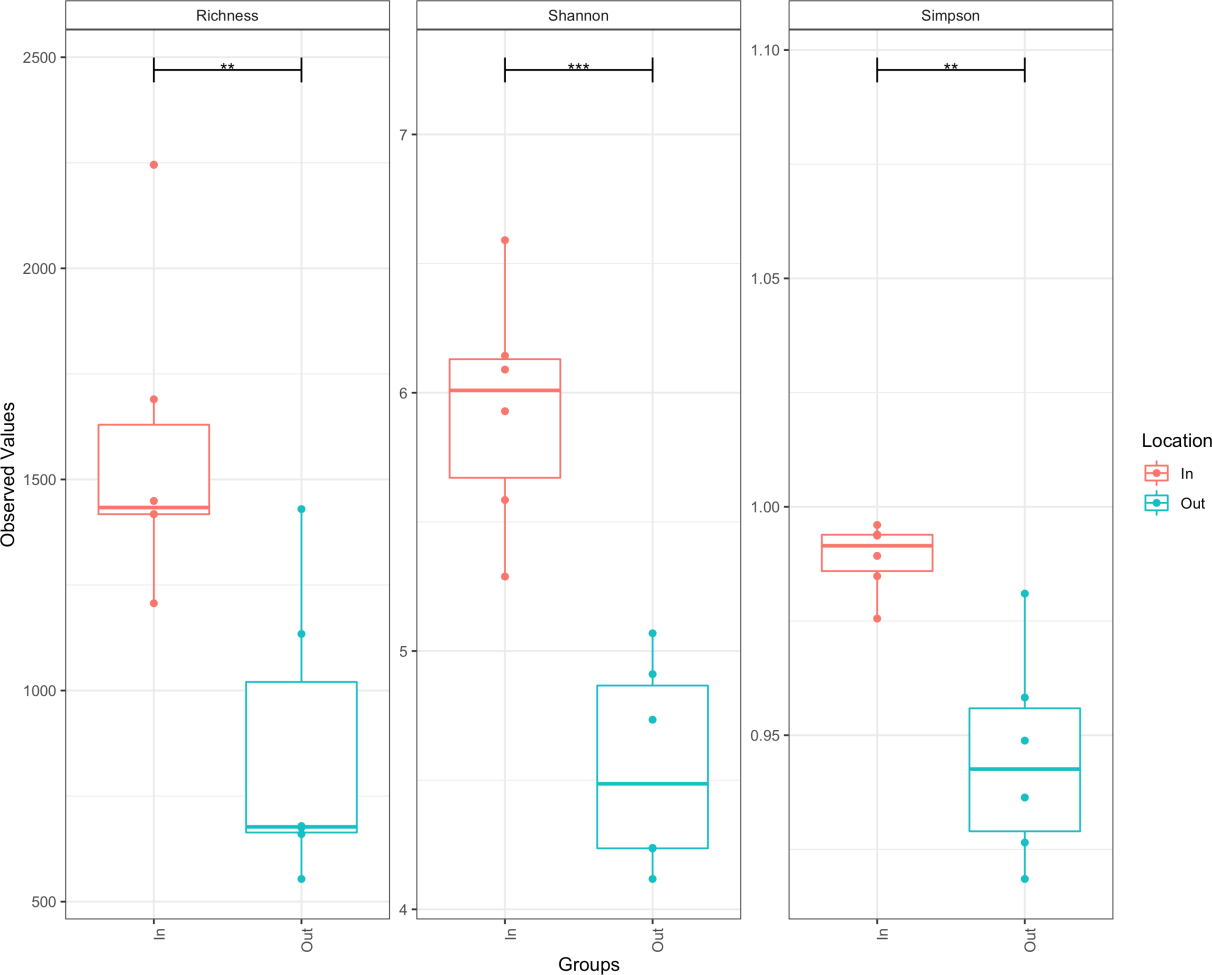
Bacterial alpha diversity measures (observed ASVs richness [Chao1], Shannon diversity, and Simpson index) of inflow (In) and outflow (Out) ponds. The line inside each box represents the median, and whiskers represent the lowest and highest levels within the 1.5 interquartile range (IQR). ** (*P* < 0.01) and *** (*P* < 0.001).

The bacterial diversity of the inflow and outflow ponds, measured in terms of the number of observed ASVs per sample (richness), Simpson index (evenness), and Shannon index, showed a significant difference in all tested alpha diversity metrics (Fig. 4). High bacterial species richness was observed in the inflow pond compared to the outflow pond (Chao 1). Very high bacterial diversity was observed in the inflow and outflow ponds (each Shannon index [H] > 3.50). However, species evenness (Simpson index, D) was slightly lower in the outflow pond than in the inflow pond. Higher Shannon and Simpson diversity indices in the inflow pond suggest a highly diverse bacterial community with relatively even species distribution. The very high Shannon and low Simpson indices of the outflow pond indicate high bacterial diversity and dominance by a few species.

**Fig 5:**
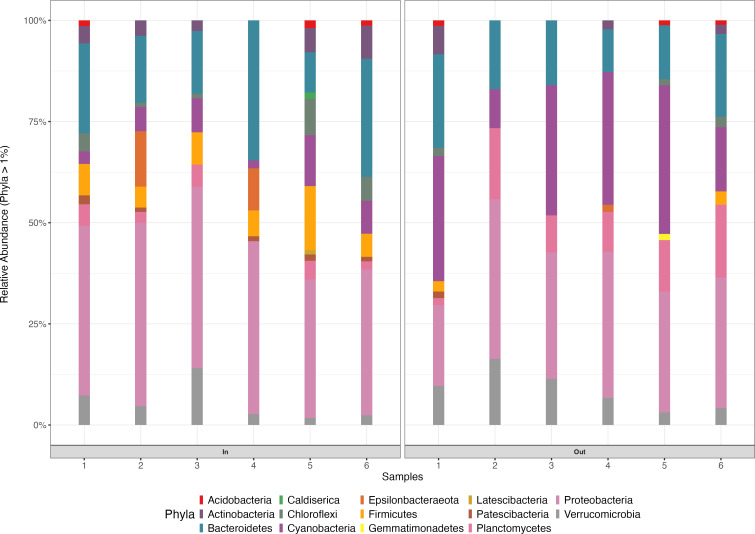
Relative abundance of bacterial taxa (phylum level) between the maturation (In, inflow; Out, outflow) pond samples. 1 to 6, inflow and outflow pond samples.

The relative abundance of bacteria at the phylum (>1%) level in all samples (*n* = 12 in total) showed the presence of 15 phyla, of which Proteobacteria (35.12% of ASVs) was the most predominant phylum across inflow and outflow samples (Fig. 5). This was followed by Bacteroidetes, Cyanobacteria, Planctomycetes, and Verrucomicrobia (18.50%, 16.13%, 7.33%, and 6.84% of ASVs, respectively). Proteobacteria was the dominant phylum as this is the fast-growing microbial community (70). This finding concurred with that of Yang et al. (71), who observed Proteobacteria as the most abundant phylum in WWTP activated sludge (AS) samples from industrial and municipal zones in China, with the highest abundance observed in locations with human interference. Bacteroidetes, Cyanobacteria, Planctomycetes, and Verrucomicrobia have also been detected in WSP (72). Bacteria belonging to the Bacteroidetes family are known to degrade organic compounds with high molecular weights (73).

**Fig 6:**
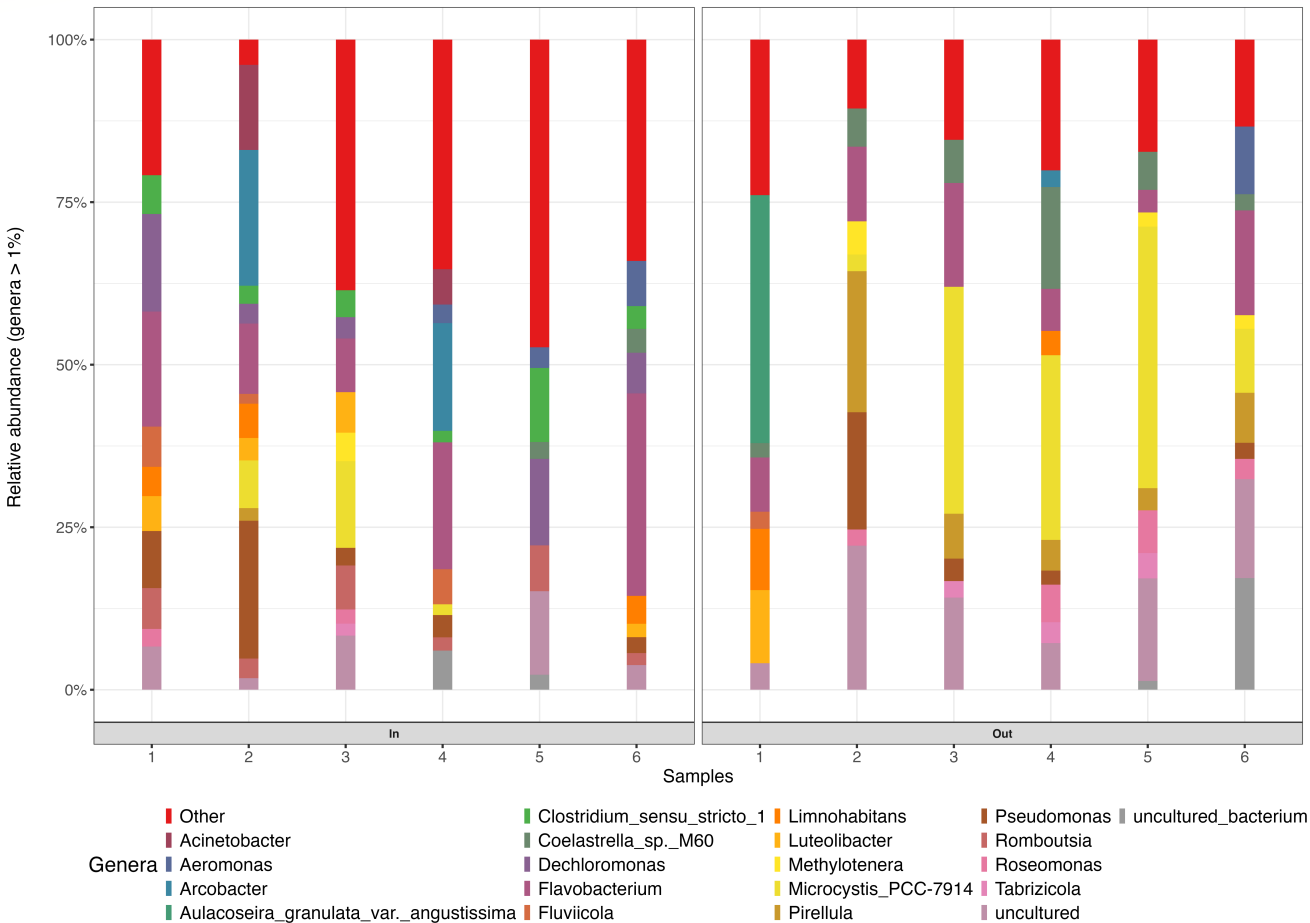
Relative abundance of the top 20 bacterial taxa (genus level) with all remaining genera grouped under “Other” between the maturation (In, inflow; Out, outflow) pond samples. 1 to 6, inflow and outflow pond samples.

While at the genus level, *Flavobacterium* and *Microcystis* were predominant (9.50% and 6.22% of ASVs, respectively), followed by *Dechloromonas* and *Pseudomonas* (4.19% and 3.56% of ASVs, respectively) across inflow and outflow pond samples (Fig. 6). There was a shift in the bacterial community of the WSP. For instance, *Dechloromonas* and *Clostridium_sensu_stricto_*1 were dominant (4.19% and 3.22% of ASVs, respectively) and were higher in the inflow pond, while *Microcystis_*PCC-7914, *Pirellula,* and *Coelastrella_*sp. *_*M60 (3.31%, 2.59%, and 1.97% of ASVs, respectively) were higher in the outflow pond than in the inflow pond. *Flavobacterium* sp. has been isolated from WWTP in Korea (74), and it was reported to degrade organic compounds in soil and water (75, 76) and paracetamol in municipal wastewater (77). While *Microcystis*, a genus of Cyanobacteria, is known to produce potent toxins, its role in degrading EPs is unknown. However, its physiological responses to such compounds can significantly influence its ecological role and the overall health of aquatic ecosystems (78). *Pseudomonas* sp. is known to degrade some emerging pollutants, including atrazine (79).

**Fig 7:**
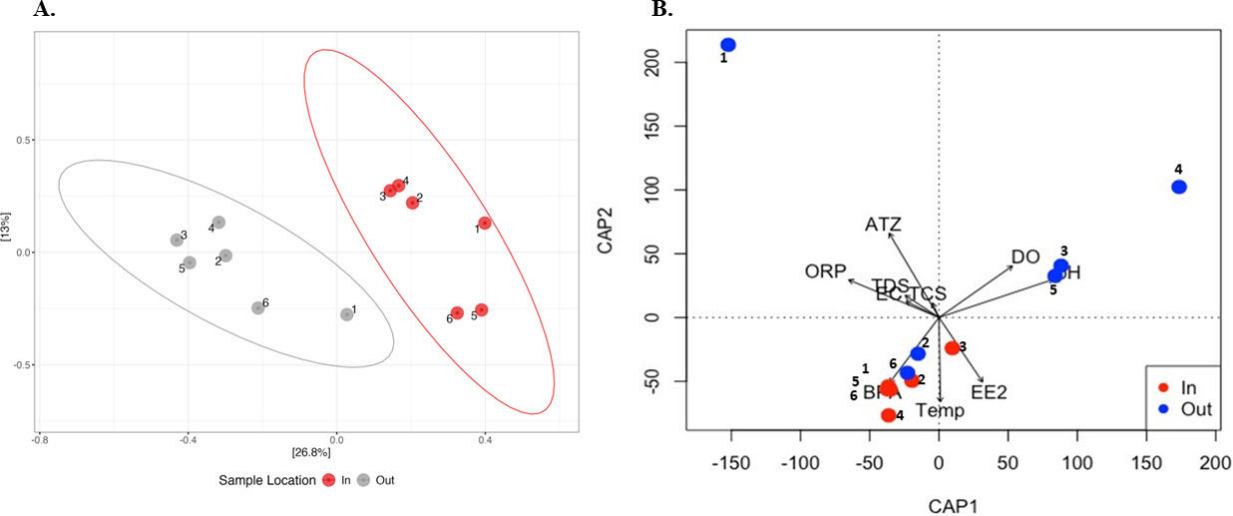
(**A**) Principal coordinate analysis plot of beta diversity of bacteria based on Bray–Curtis dissimilarities at the ASV level of the WSP. The PCoA data revealed that the first two PCoA components explained PC1 (26.8%) and PC2 (13%) of the variation, respectively. Red dot represents all the samples collected from the inflow pond; gray dot represents all the samples collected from the outflow pond; 1 to 6, sample names. (**B**) Distance-based redundancy analysis (db-RDA) biplot of WSP for bacterial community. The first two constrained axes accounted for CAP1 (51%) and CAP2 (29%) of the explained variance, respectively. Red dots, inflow pond; blue dots, outflow pond; 1 to 6, sample names. The arrows indicate physicochemical parameters (ORP, DO, TDS, pH, EC, temp, TCS, BPA, EE2, and ATZ) of the inflow and outflow ponds.

The principal coordinate analysis revealed that the clustering of the WSP samples was primarily influenced by their locations. (Fig. 7A). Distinct bacterial communities were detected between the inflow and outflow ponds (PERMANOVA F_1,11_ = 3.12, R^2^ = 24%, and *P* = 0.002)

The distance-based redundancy analysis (db-RDA) was used to explore the relationship between bacterial community structure and environmental (physicochemical) variables across inflow and outflow samples from the maturation ponds (Fig. 7B). Although the constrained axes (CAP1 and CAP2) together account for a large proportion of the explained variation, the overall model was not statistically significant (adjusted R² = 0.28, *P* = 0.44), suggesting limited predictive power of the environmental variables in explaining community composition.

The ordination also shows that sample 1 (Out) aligns closely with ATZ and ORP, while sample 4 (Out) is positively associated with DO and pH. Several inflow samples cluster together and appear to be moderately influenced by EE2, BPA, and temperature. These results suggest that while environmental variables collectively explain some variation in community structure, pH may be the most influential individual factor in shaping microbial composition in the maturation ponds. To evaluate the relationship between prokaryotic community composition and environmental variables, a PERMANOVA was performed with pH, electrical conductivity (EC), and total dissolved solids (TDS) included as continuous predictors in a multivariate regression framework based on Bray–Curtis dissimilarities. This analysis was conducted independently of the db-RDA and should be interpreted as testing the strength of association between individual environmental variables and community composition, rather than as a constrained ordination. The PERMANOVA results indicated that pH (PERMANOVA F1,11 = 3.05, R2 = 23%, and *P* = 0.001) was significantly associated with variation in prokaryotic community structure.

### Eukaryotic diversity and abundance

A total of 878 eukaryotic ASVs (>99% similarity cut-off), ranging from 21 to 183 ASVs per sample, were found using identical sequencing depth in all samples. Of the total number of ASVs, 1,836 (representing 16.09% of the total number of sequences) were unique to inflow samples, 253 (1.09%) were unique to outflow samples, and 351 (82.82%) were shared between all the locations.

**Fig 8:**
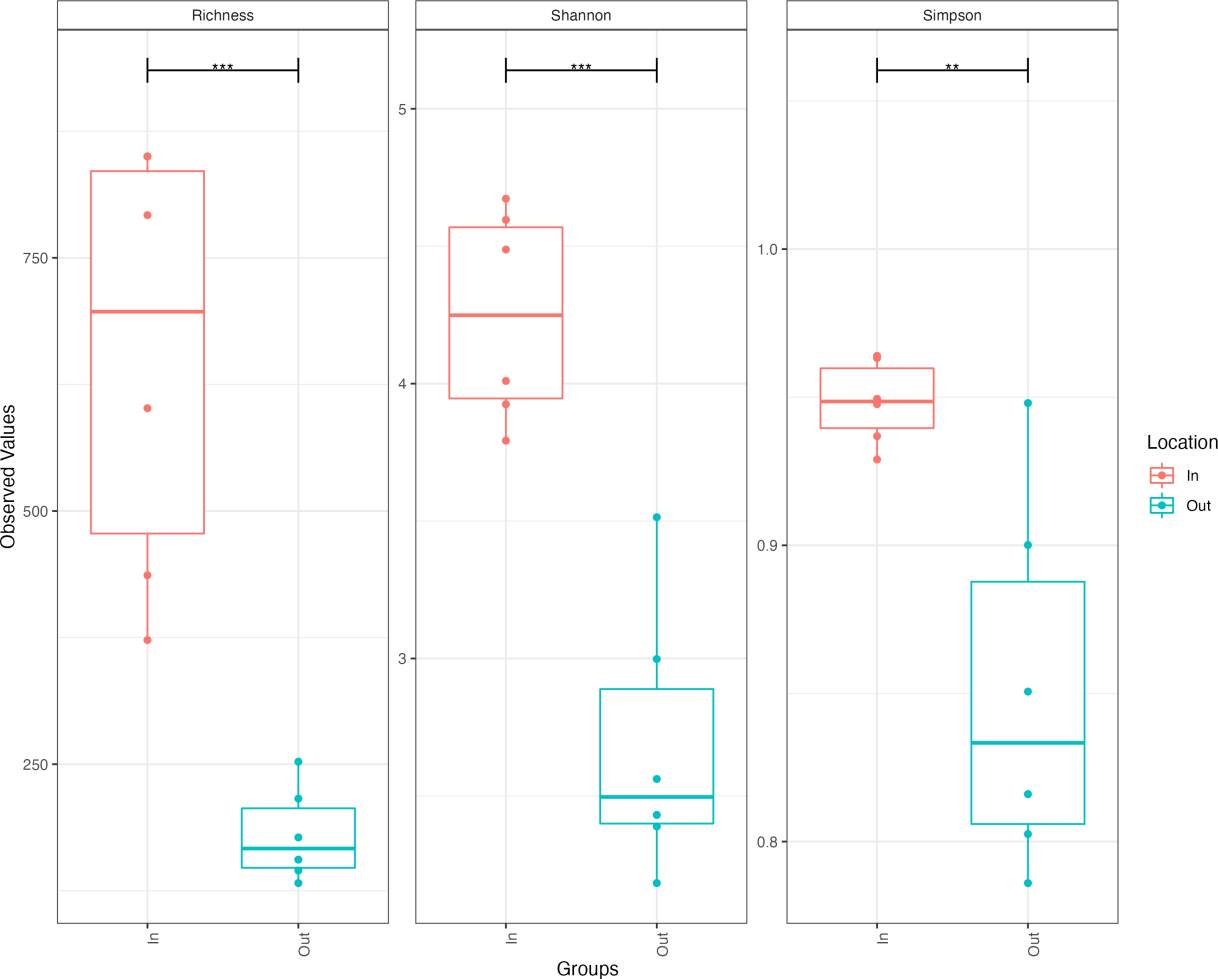
Eukaryotic alpha diversity measures (observed number of ASV richness for Chao1, Shannon diversity, and Simpson index) of inflow (In) and outflow (Out) ponds. The line inside each box represents the median, and whiskers represent the lowest and highest levels within the 1.5 interquartile range (IQR). ** (*P* < 0.01) and *** (*P* < 0.001).

The eukaryotic diversity between inflow and outflow ponds, measured in terms of the number of observed ASVs per sample (richness), Simpson index (evenness), and Shannon index, showed a significant difference in alpha diversity (Fig. 8). High eukaryotic species richness was observed in the inflow pond compared to the outflow pond (Chao 1), and there was very high eukaryotic diversity in the inflow pond (Shannon index, H > 3.50), while moderate (median H = 2.50) diversity was observed in the outflow pond. However, eukaryotic species evenness (Simpson index, D) was slightly lower in the outflow pond than in the inflow pond. Higher Shannon and Simpson diversity indices in the inflow pond suggest a highly diverse eukaryotic community with a relatively even eukaryotic species distribution. Meanwhile, moderate Shannon and low Simpson indices in the outflow pond indicate moderate eukaryotic diversity and dominance by a few species.

**Fig 9:**
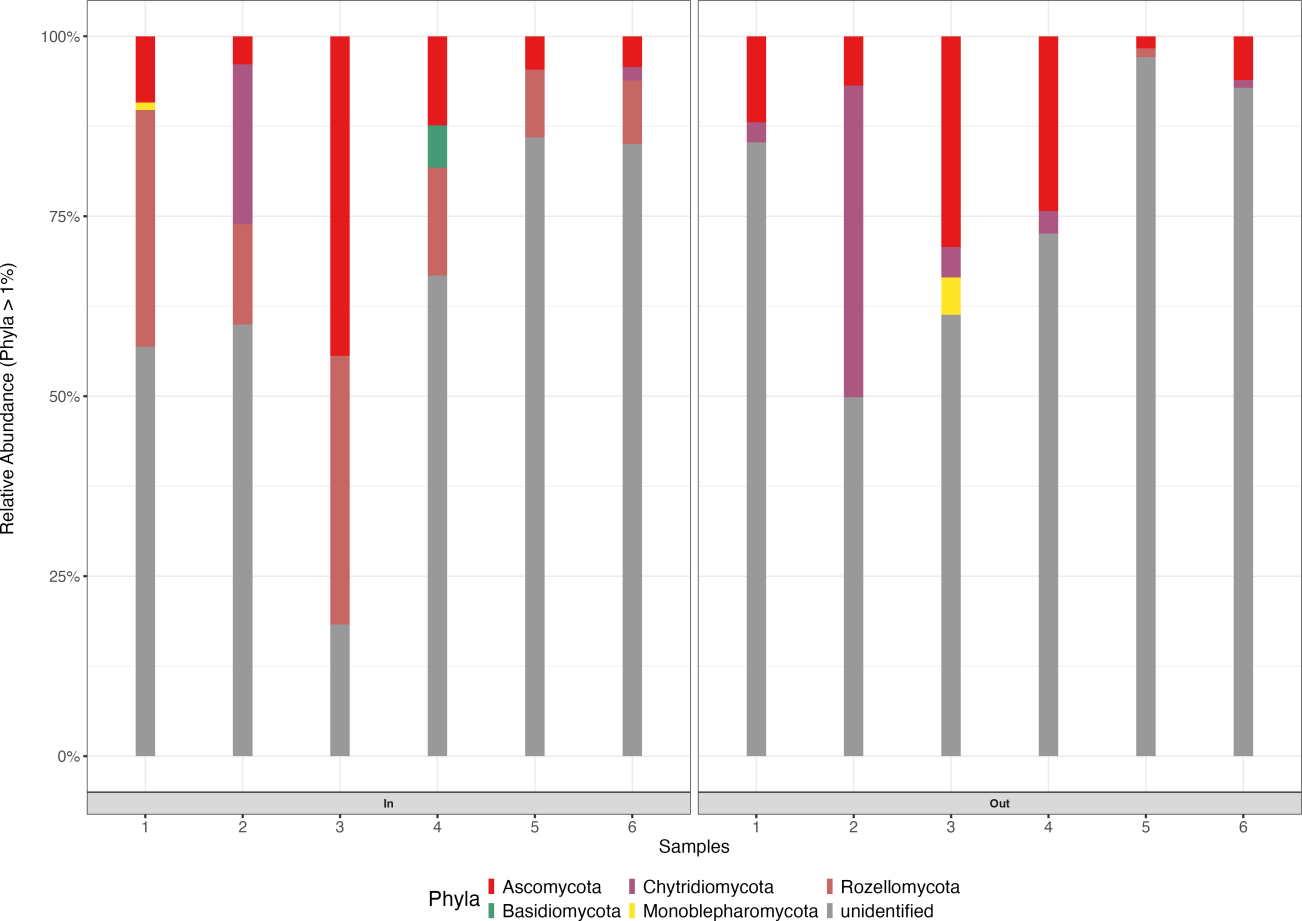
Relative abundance of eukaryotic taxa (phylum level) between the maturation (In, inflow; Out, outflow) pond samples. 1 to 6, inflow and outflow pond samples.

The taxonomically assigned ASVs revealed that members of the WSP eukaryotic communities were mainly assigned to Ascomycota, Chytridiomycota, Rozellomycota, Basidiomycota, and Monoblepharomycota. The unidentified phyla have no reference sequences available in the UNITE database (80). The relative abundance of eukaryotes at the phylum level (>1%) showed the predominance of unidentified organisms (68.85% of ASVs) followed by Ascomycota (13.14% of ASVs) across inflow and outflow samples (Fig. 9). High unidentified eukaryotes highlight a gap in current databases and limit the ecological interpretation of the eukaryotic role. There was a shift in the eukaryotic population of the WSP pond; the Rozellomycota population in the inflow pond (9.66% of ASVs) drastically reduced in the outflow pond (0.27% of ASVs), similar to Basidiomycota (inflow: 0.69% and outflow: 0.07% of ASVs). However, the Chytridiomycota population increased in the outflow pond (inflow: 2.07% and outflow: 4.55% of ASVs) (81, 82). Most of the core eukaryotic phyla identified in this study, Ascomycota, Basidiomycota, Chytridiomycota, and Rozellomycota, have been frequently reported in WWTP environments. Among these, Ascomycota and Basidiomycota are typically the most prevalent due to their metabolic versatility and capacity to adapt to diverse environmental conditions (83). Chytridiomycota and Rozellomycota are generally less abundant, are consistently detected, and may fulfill specialized ecological functions (84). Notably, Rozellomycota has been associated with high organic load environments in WWTPs, suggesting a potential role in organic matter degradation. Furthermore, Rozellomycota is especially prevalent in aquatic habitats, where it is thought to thrive under nutrient-rich conditions. Importantly, Chytridiomycota and Rozellomycota are part of a group of largely uncharacterized eukaryotic lineages, often referred to as “dark matter fungi” (85). These basal eukaryotic phyla remain poorly understood, yet they represent a significant portion of eukaryotic diversity in aquatic and engineered ecosystems. Previous studies have corroborated the presence of these phyla in WWTPs, with Niu et al. (2017) detecting Chytridiomycota, Ascomycota, and Basidiomycota and (85) identifying Chytridiomycota and Rozellomycota within such systems.

**Fig 10:**
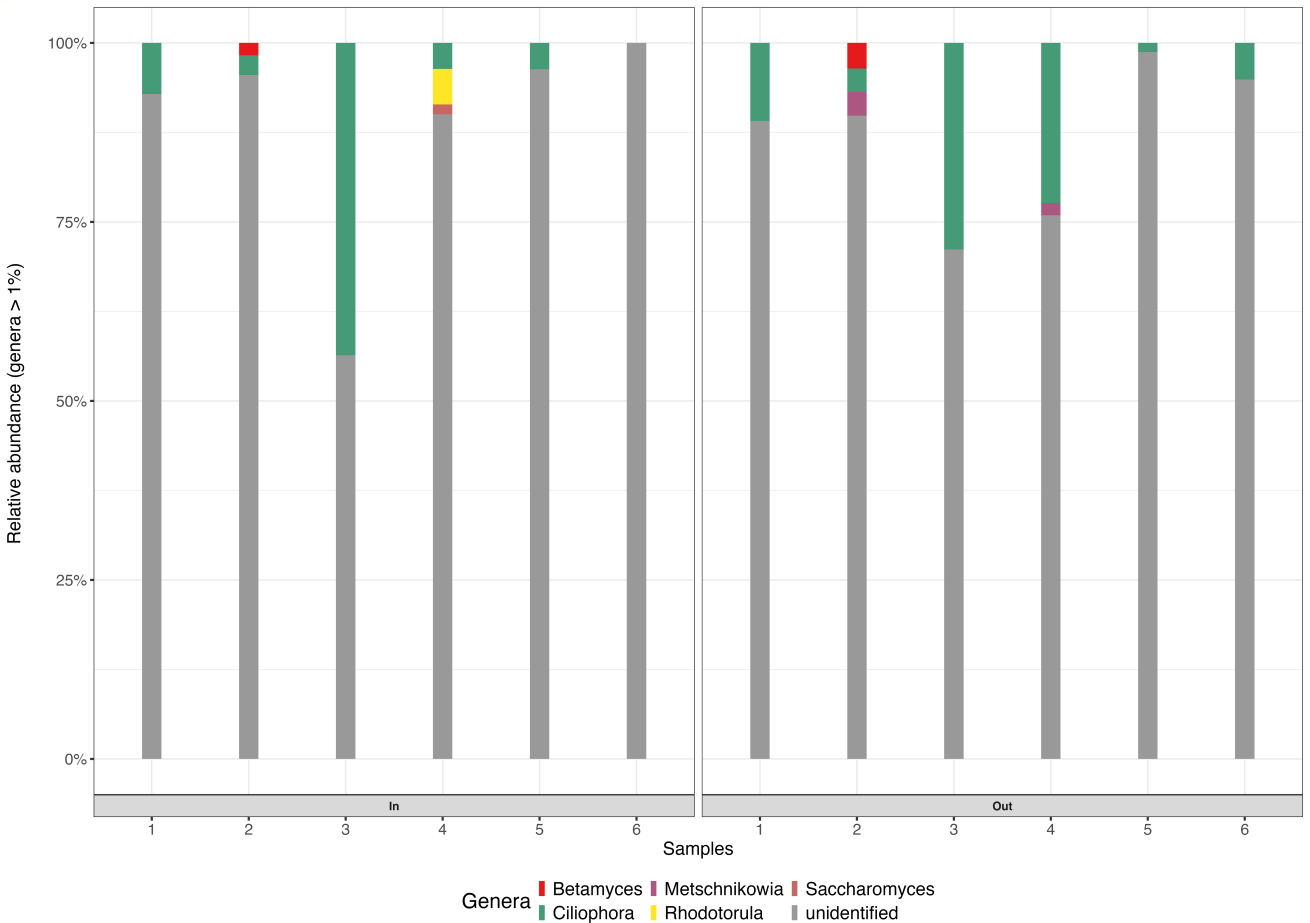
Relative abundance of eukaryotic taxa at the genus level between the maturation (In, inflow; Out, outflow) pond samples. 1 to 6, inflow and outflow pond samples.

Most eukaryotic ASVs remained taxonomically unclassified at the genus level, with unidentified organisms accounting for 85.88% of the total ASVs (Fig. 10). Among the classified taxa, sequences assigned to the phylum *Ciliophora* constituted 10.86% of ASVs. While *Ciliophora* is a well-established phylum within the domain Eukaryota, variously classified under Protista, Protozoa, or Chromista, the sequences detected in this study may represent a novel eukaryotic genus closely affiliated with *Ciliophora*, but not yet represented in current reference databases. The detection of *Ciliophora* further emphasizes the broad eukaryotic coverage of the ITS marker beyond fungi. There was a slight shift in the eukaryotic population. The *Betamyces* and *Metschnikowia* populations were higher in the outflow pond (0.36% and 0.57%) than inflow (0.14% and 0.06%), respectively. However, *Rhodotorula* sp. and *Saccharomyces* sp. populations were higher in the inflow (0.40% and 0.19%) than in the outflow pond (0.2% and 0.00%), respectively. *Rhodotorula* sp. has been reported to degrade phenolic compounds (86), while *Saccharomyces* sp. removed cadmium ion (Cd^2+^) from wastewater (87).

**Fig 11:**
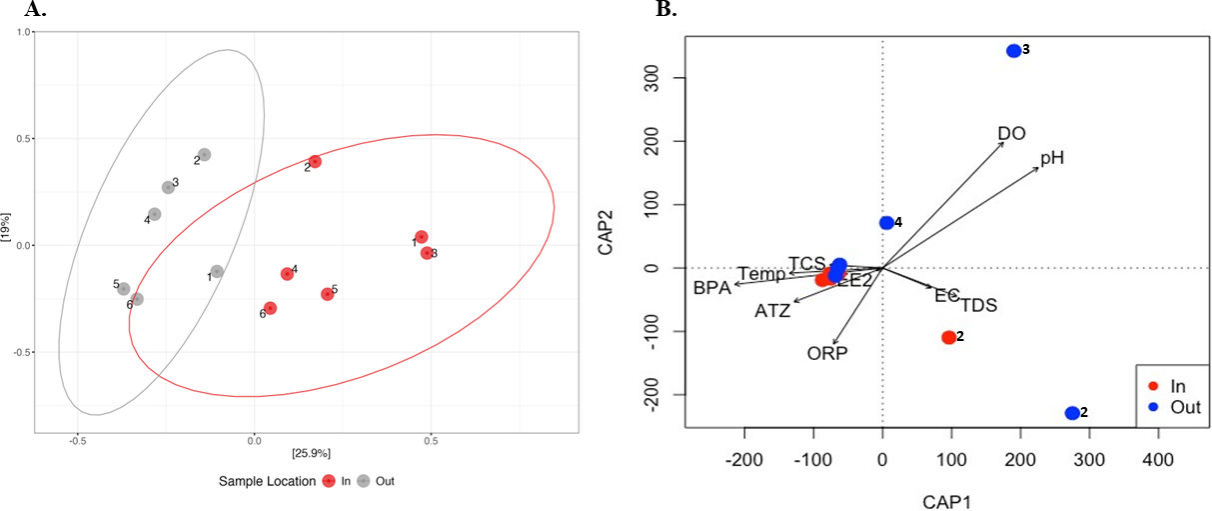
(**A**) Principal coordinate analysis plot of beta diversity of eukaryotic communities based on Bray–Curtis dissimilarities at the ASV level of the WSP. The PCoA data revealed that the first two PCoA components explained PC1 (23.9%) and PC2 (17.4%) of the variation, respectively. Red dot represents all the samples collected from the inflow pond; gray dot represents all the samples collected from the outflow pond; 1 to 6, inflow and outflow pond samples. (**B**) Distance-based redundancy analysis (db-RDA) biplot of WSP for eukaryotic communities. The first two constrained axes accounted for CAP1 (47%) and CAP2 (30%) of the explained variance, respectively. Red dot, inflow pond; blue dot, outflow pond; 1 to 6, inflow and outflow pond samples. The arrows indicate the physicochemical parameters (ORP, DO, TDS, pH, EC, temp, TCS, BPA, EE2, and ATZ) of the inflow and outflow ponds.

The principal coordinate analysis revealed location-based clustering of WSP samples (Fig. 11A). Distinct eukaryotic communities were detected between the inflow and outflow ponds (PERMANOVA F_1,11_ = 2.78, R^2^ = 22%, and *P* = 0.001), indicating that the location had a significant effect on eukaryotic diversity.

The distance-based redundancy analysis (db-RDA) was used to investigate the association between eukaryotic community structure and environmental parameters across inflow and outflow samples in the maturation ponds (Fig. 11B). The first two constrained axes (CAP1 and CAP2) together explained approximately 77% of the variation in community composition. However, the overall db-RDA model was not statistically significant (adjusted R² = 0.37, *P* = 0.33), indicating limited explanatory power of the environmental variables.

The ordination plot indicates that sample 2 is aligned along the direction of the TDS and EC vectors, suggesting a possible association. Most inflow samples appeared more tightly clustered and were more influenced by BPA, ATZ, ORP, and temperature. Outflow samples showed a greater spread, with samples 3 and 4 showing alignment with DO and pH, respectively. These patterns suggest that, while overall environmental influence was weak, specific factors, such as TDS, may contribute to structuring eukaryotic communities under localized conditions. To evaluate the relationship between eukaryotic community composition and environmental variables, a PERMANOVA was performed with pH, electrical conductivity (EC), and total dissolved solids (TDS) included as continuous predictors in a multivariate regression framework based on Bray–Curtis dissimilarities. This analysis was conducted independently of the db-RDA and should be interpreted as testing the strength of association between individual environmental variables and community composition, rather than as a constrained ordination. The PERMANOVA results indicated that pH (PERMANOVA F1,11 = 2.48, R2 = 20%, and *P* = 0.001), EC (PERMANOVA F1,11 = 1.75, R2 = 15%, and *P* = 0.028), and TDS (PERMANOVA F1,11 = 1.87, R2 = 16%, and *P* = 0.009) were significantly associated with variation in eukaryotic community structure.

Functional analysis can provide a deeper understanding of how microbial communities support treatment processes as it reveals the potential functions of these communities within a given environment. Using PICRUSt2, a well-known bioinformatics tool, the metabolic capabilities of bacterial communities based on 16S rRNA gene sequencing data could be predicted (88). Functional analysis is useful in wastewater systems, where both unidentified and known microorganisms are present, as well as in the presence of contaminants. Focusing on possible microbial metabolic activities, the predictive functional profiling could suggest the presence of biomarkers for degrading pollutants.

### Functional analysis

**Fig 12:**
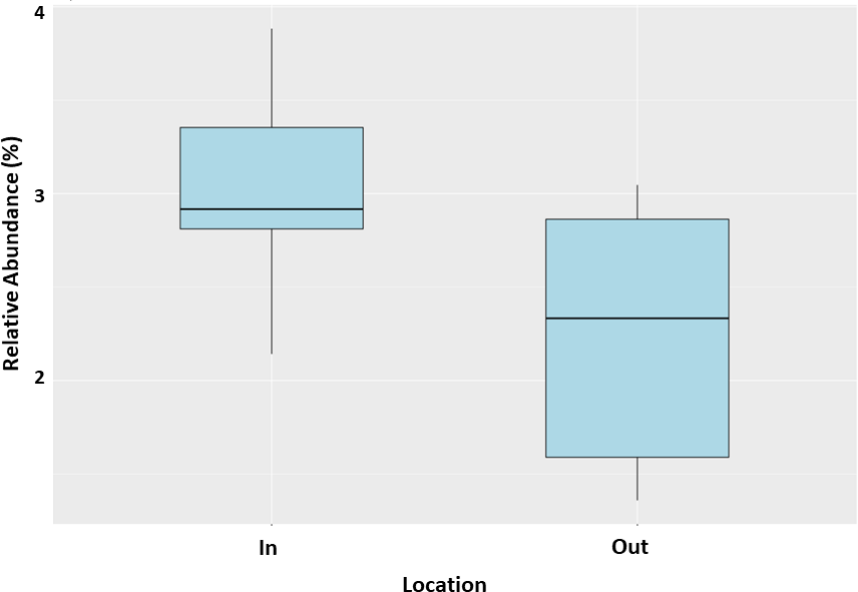
Boxplot of PICRUSt2 energy metabolism-associated KEGG pathways based on the mean proportions (*P* < 0.05) and identities of 16S rRNA genes in WSP ponds during the sampling period between the inflow (In) and outflow (Out) ponds.

PICRUSt2 was used to predict the presence of functional pathways related to the WSP maturation pond system. A total of 93,996 Kyoto Encyclopedia of Genes and Genomes (KEGG) orthologs were obtained from the PICRUSt2 analysis. Among them, 18,877 orthologs had zero KO hits in all samples. Furthermore, 6,573 KEGG orthologs had KO hits of 500 or more in at least one sample out of the 12 WSP samples. The orthologs were identified and grouped according to KEGG functional pathways and Gene Ontology affiliation, which included xenobiotics, carbohydrates, and energy metabolism. The energy metabolism was predicted to be significantly higher in the bacterial community of the inflow pond of the WSP (*P* <0.05) (Fig. 12).

**Fig 13:**
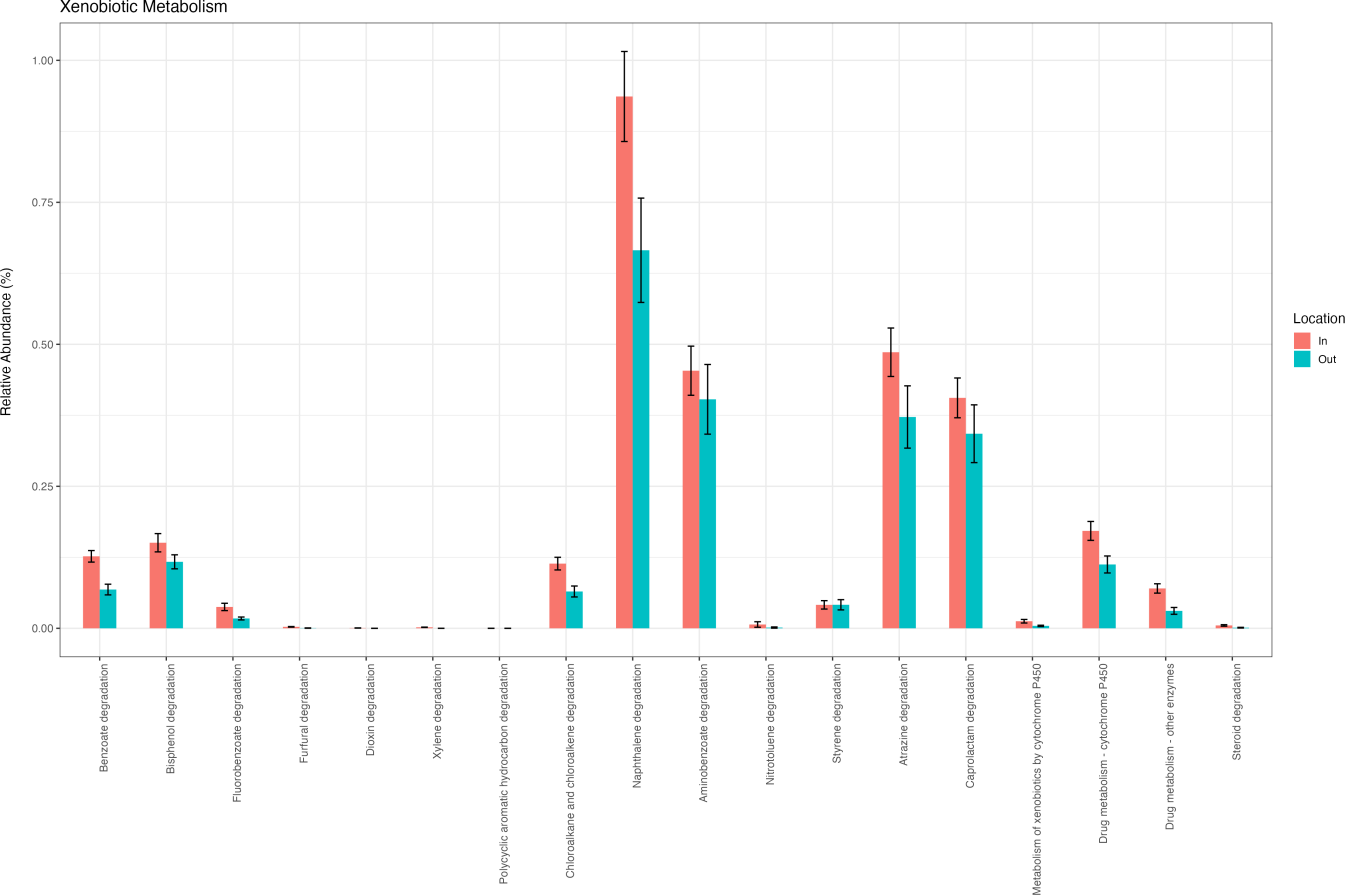
Predicted functional xenobiotic metabolism pathways of the WSP maturation ponds (inflow pond [red] and outflow pond [blue]) inferred in the KEGG pathway database from the 16S rRNA gene sequences using PICRUSt2.

The KEGG pathway database was used to analyze the biodegradation pathways of contaminants. Using PICRUSt2, 18 KEGG-specific metabolic sub-pathways involved in xenobiotic degradation were present in WSP (inflow and outflow) (Fig. 13). Of these xenobiotic degradation pathways, the atrazine degradation pathway was present, and three genes (*ureA, B,* and *C*; urease sub-units [EC: 3.5.1.5]; *atzF*; allophanate hydrolase [EC:3.5.1.54] and urea carboxylase [EC:6.3.4.6]) were more abundant than *atzD*; cyanuric acid amidohydrolase [EC:3.5.2.15] between the ponds. The gene combination of *trzN/atzA-atzB, C* leads to the transformation of atrazine to its intermediate cyanuric acid. It was reported that the presence of the *trzN*, *atzC,* and *trzD* genes indicates atrazine degradation via the hydroxy-atrazine pathway (*trzN*) to cyanuric (*atzC*), followed by biuret, NH_3_, and CO_2_ (*trzD*) (89). However, the presence of genes does not imply that the genes are functional; this could be why atrazine accumulated in the outflow pond.

The naphthalene degradation pathway is prominent in the predicted functional xenobiotic metabolism pathways (Fig. 13), indicating the possible involvement of the bacterial community in naphthalene degradation. In addition, pathways for aminobenzoate, bisphenol, atrazine, and caprolactam degradation were also present.

The BPA degradation pathway involves metabolic pathways for various types of compounds, including benzoates, chlorocyclohexane, chlorobenzene, polycyclic aromatic hydrocarbons, xylene, and drug metabolism, specifically cytochrome P450 degradation (90). Three genes present in the BPA biodegradation pathway include E1.13.11.41; 2,4′-dihydroxyacetophenone dioxygenase [EC:1.13.11.41], *hapE*; 4-hydroxyacetophenone monooxygenase [EC:1.14.13.84], and *PON*; arylesterase/paraoxonase [EC:3.1.1.2, and EC:3.1.8.1]. Yu et al. (91) reported that the degradation pathway predicted was based on the known paired relationship between genes, substrates, and products in the KEGG database.

Though the degradation pathway of EE2 has not been documented in the KEGG database, EE2 can be degraded to estrone (E1), a steroid hormone (92). Nine genes associated with the steroid degradation pathway are present, including *choD* (cholesterol oxidase) [EC:1.1.3.6].

Triclosan (TCS) biodegradation pathways remain incompletely elucidated and have not yet been incorporated into the KEGG database. Nevertheless, research indicates that triclosan degradation involves diverse metabolic processes and enzymatic activities similar to those observed for other xenobiotics. Notably, several studies have identified TcsA, an osmolarity two-component system sensor histidine kinase [EC:2.7.13.3], in *Sphingomonas* sp., which is associated with triclosan degradation (93, 94). This highlights the need for further investigation into the genetic and enzymatic mechanisms underlying triclosan degradation to facilitate its inclusion in metabolic pathway databases.

### Conclusion

This study emphasizes the crucial importance of monitoring emerging pollutants (EPs) and microbial communities within wastewater maturation pond systems, particularly in regions where these systems are still in use. Among the four targeted compounds, bisphenol A (BPA), atrazine (ATZ), triclosan (TCS), and 17α-ethinylestradiol (EE2), ATZ and EE2 showed notable persistence in WSPs. The selected wastewater treatment plant (WWTP) is the only facility in Bloemfontein that still operates a series of wastewater stabilization or maturation ponds as part of its treatment system. The persistence of endocrine-disrupting chemicals (EDCs) despite biological treatment underscores the limitations of current wastewater management practices and signals the urgent need for more effective, context-specific remediation strategies. Using 16S rRNA and ITS sequencing, the study profiled bacterial and eukaryotic communities across WSP influent and effluent ponds. Notably, Proteobacteria dominated bacterial communities throughout the system, with increased representation of *Flavobacterium*, *Microcystis, Dechloromonas,* and *Pseudomonas* in effluent sites, genera previously associated with EP degradation, including atrazine and bisphenol A. Eukaryotic communities were more variable, with the phyla Ascomycota, Chytridiomycota, Rozellomycota, Basidiomycota, and Monoblepharomycota. A striking observation was the high relative abundance (~30%) of unidentified eukaryotic ASVs, indicating a potentially underexplored reservoir of eukaryotic biodegraders in WSP systems.

Multivariate analyses, including db-RDA, revealed that pH and TDS were the most significant environmental variables shaping microbial compositions. Clustering of WSP samples into distinct positions, as revealed by PCoA, showed microbial communities were grouped according to their WSP sample location (In and Out). Bacterial functional prediction using PICRUSt2 and KEGG pathway analysis suggested an enrichment of genes associated with xenobiotic degradation pathways, including those involved in the metabolism of naphthalene, atrazine, and caprolactam. These predicted functions were predominantly observed in microbial communities from the influent ponds, aligning with the elevated relative abundance of known xenobiotic degraders such as *Pseudomonas* and *Acinetobacter* sp. This suggests that these taxa may play an active role in the initial transformation of EPs within the wastewater system. Although the identification of key EDC degradation genes highlights potential metabolic capabilities, experimental validation would be necessary to confirm active degradation. These findings suggest that low-energy systems, such as wastewater maturation ponds, support diverse and functionally relevant microbial communities with a limited yet measurable capacity for EP attenuation. The persistence of EPs, alongside the high abundance of unidentified eukaryotic taxa, underscores the need for targeted interventions, such as bioaugmentation or biostimulation processes, to enhance microbial degradation potential. Maturation ponds provide a cost-effective and low-energy approach for tertiary wastewater treatment, yet their performance in removing EPs such as EDCs can vary with environmental and microbial factors. The findings suggest that indigenous microbial communities possess the genetic potential for EDC degradation, which could be harnessed to improve system performance. To translate these insights into management practices, several strategies can be considered: (i) adjusting hydraulic retention times and aeration regimes to maintain conditions favorable for EDC-degrading taxa; (ii) bioaugmentation with microbial strains carrying key functional genes (e.g., monooxygenase, laccase, or dioxygenase) identified in this study; and (iii) establishing molecular monitoring frameworks (qPCR and meta-transcriptomic screening) for early detection of EDC accumulation or functional gene loss. Together, these actions could strengthen the resilience and pollutant removal efficiency of maturation pond systems, aligning with sustainable wastewater management goals.

## Data Availability

Data are available within the article and its supplemental material. Amplicon sequencing reads (16S rRNA and ITS genes) are available at the National Center for Biotechnology Information (NCBI) Sequence Read Archive (SRA) database. The accession number for all bacterial and eukaryotic sequences deposited into the NCBI SRA database is PRJNA1234587.

## SUPPLEMENTAL MATERIAL

Table S1 aem.01342-25-s0001.docx MRM transitions per analyte used for LC-MS/MS analysis.

Table S2 aem.01342-25-s0002.docx Physicochemical parameters of maturation ponds.

ASM does not own the copyrights to Supplemental Material that may be linked to, or accessed through, an article. The authors have granted ASM a non-exclusive, world-wide license to publish the Supplemental Material files. Please contact the corresponding author directly for reuse.
