# Supplementary material for: Monitoring endocrine-disrupting chemicals and microbial diversity of wastewater treatment maturation ponds
Source: Appl Environ Microbiol. 2026 Apr 29;92(5):e01342-25. doi: 10.1128/aem.01342-25 (PMC13188910; doi:10.1128/aem.01342-25)
Supplement: Table S1 — MRM transitions per analyte used for LC-MS/MS analysis. [file aem.01342-25-s0001.docx]

1. *The MRM (Multiple Reaction Monitoring) transitions per analyte used for the LC-MS/MS analysis.*

The peak area on the chromatograph generated from the first and most sensitive transition was used as the quantifier, while the second transition was used as a qualifier. The qualified one serves as an additional level of confirmation for the presence of the analytes; the retention time for these two transitions should be the same. The peak areas of the unknown samples were related to a quantified value using the calibration curve for each analyte, normalised to the peak area of the internal standard (Supplementary Table 1).

**Supplementary Table S1:** List of MRM transitions for each LC-MS/MS analyte.

| **Analyte** | **Ionization mode** | **Column** | **Solvent A** | **Solvent B** | **Q1** | **Q3** |  |
| --- | --- | --- | --- | --- | --- | --- | --- |
| Atrazine | Positive | Gemini NX, 150 mm x 4.6 mm, Phenomenex | H_2_O/0.1% formic Acid | Acetonitrile/0.1% formic acid | 216.2 | 174.0 | Quantifier |
|  |  |  |  |  | 216.2 | 68.1 | Qualifier |
| Triclosan | Negative | Kinetix Biphenyl 150 mm x 4.6 mm, Phenomenex | H_2_O/0.1% formic Acid | Acetonitrile/0.1% formic acid | 286.0 | 35.0 | Quantifier |
|  |  |  |  |  | 286.0 | 241.2 | Qualifier |
| EE2 | Negative | Gemini NX, 150 mm x 4. 6mm, Phenomenex | H_2_O/0.1% NH_4_OH | Acetonitrile/0.1% NH_4_OH | 287.0 | 35.0 | Quantifier |
|  |  |  |  |  | 287.0 | 241.2 | Qualifier |
| BPA | Negative | Gemini NX, 150 mm x 4.6 mm, Phenomenex | H_2_O/0.1% NH_4_OH | Acetonitrile/0.1% NH_4_OH | 227.1 | 133.0 | Quantifier |
|  |  |  |  |  | 227.1 | 93.0 | Qualifier |
