# Supplementary material for: Monitoring endocrine-disrupting chemicals and microbial diversity of wastewater treatment maturation ponds
Source: Appl Environ Microbiol. 2026 Apr 29;92(5):e01342-25. doi: 10.1128/aem.01342-25 (PMC13188910; doi:10.1128/aem.01342-25)
Supplement: Table S2 — Physicochemical parameters of maturation ponds. [file aem.01342-25-s0002.docx]

**2.** *Physicochemical parameters of maturation ponds*

Supplementary Table S2 presents the results of physicochemical analysis conducted during the six-month sampling period (September to February).

| **Sampling sites** | **Seasons** | **Dates** | **Parameters (Mean ± SD)** | | | | | |  |
| --- | --- | --- | --- | --- | --- | --- | --- | --- | --- |
|  |  |  | **pH** | **EC (µS/cm)** | **TDS (mg/L)** | **DO (mg/L)** | **ORP (mV)** | **Temperature (°C)** |  |
|  |  |  |  |  |  |  |  |  |  |
| **Inflow Pond (P1)** | Spring | Sept | 7,40 ± 0,15 | 688,00 ± 0,82 | 550,30 ± 0,58 | 4,67 ± 0,02 | 177,00 ± 5,46 | 20,20 ± 0,11 |  |
|  |  | Oct | 7,70 ± 0,13 | 712,30 ± 1,70 | 569,70 ± 1,53 | 1,59 ± 0,03 | 156,30 ± 5,06 | 21,40 ± 0,18 |  |
|  |  | Nov | 7,60 ± 0,01 | 689,70 ± 2,36 | 551,30 ± 2,31 | 3,48 ± 0,59 | 201,90 ± 9,82 | 24,00 ± 0,23 |  |
|  | Summer | Dec | 7,80 ± 0,10 | 747,00 ± 2,16 | 560,00 ± 2,65 | 8,11 ± 0,71 | 115,40 ± 4,09 | 25,80 ± 0,91 |  |
|  |  | Jan | 8,30 ± 0,29 | 706,30 ± 6,94 | 529,70 ± 6,43 | 15,77 ± 2,14 | 113,00 ± 19,50 | 27,80 ± 0,49 |  |
|  |  | Feb | 8,20 ± 0,12 | 507,30 ± 1,70 | 380,70 ± 1,53 | 10,31 ± 0,52 | 105,30 ± 6,50 | 31,90 ± 1,03 |  |
| **Outflow Pond (P2)** | Spring | Sept | 7,80 ± 0,07 | 723,30 ± 1,70 | 578,70 ± 1,53 | 8,83 ± 0,06 | 202,20 ± 2,46 | 18,30 ± 0,10 |  |
|  |  | Oct | 9,70 ± 0,05 | 646,30 ± 0,47 | 532,00 ± 25,12 | 13,31 ± 0,26 | 134,60 ± 3,76 | 21,70 ± 0,15 |  |
|  |  | Nov | 10,10 ± 0,00 | 625,70 ± 0,94 | 500,70 ± 1,15 | 17,49 ± 0,42 | 117,70 ± 5,64 | 22,20 ± 0,27 |  |
|  | Summer | Dec | 9,30 ± 0,03 | 713,30 ± 8,99 | 535,30 ± 8,02 | 12,19 ± 0,44 | 89,60 ± 2,45 | 21,10 ± 0,22 |  |
|  |  | Jan | 10,40 ± 0,09 | 382,30 ± 8,26 | 287,00 ± 7,81 | 12,54 ± 1,66 | 94,70 ± 7,63 | 23,60 ± 0,72 |  |
|  |  | Feb | 9,40 ± 0,17 | 290,00 ± 2,94 | 217,70 ± 2,52 | 1,38 ± 0,42 | 136,30 ± 3,68 | 22,30 ± 0,26 |  |
